# Supplementary material for: Examining the flavor descriptors of e-cigarettes, heated tobacco products, and nicotine pouches in the Philippines: Regulatory challenges and opportunities
Source: PLOS Glob Public Health. 2025 Feb 13;5(2):e0004248. doi: 10.1371/journal.pgph.0004248 (PMC11824988; doi:10.1371/journal.pgph.0004248)
Supplement: S2 Table — (DOCX) [file pgph.0004248.s002.docx]

# **Table 2. Definition of Terms**

*All terms below are defined by Republic Act No. 11900 or the “Vaporized Nicotine and Non-Nicotine Products Regulation Act.”*

| **Product Type** | **Definition** |
| --- | --- |
| **Vapor Products, also referred to as Vapor Product Refills** | Refers to the liquid, solid, or gel, or any combination thereof, which may or may not contain nicotine, that is transformed into an aerosol without combustion by a Vapor Product Device. |
| **Vapor Product Device** | Refers to a device or a combination of devices used to heat a Vapor Product, to produce an aerosol, mist, or vapor that users inhale. These may include combinations of a liquid solution or gel that are heated and transformed into an aerosol without combustion through the employment of a mechanical or electronic heating element, battery, or circuit, and includes, but is not limited to, a cartridge, a tank, or a device without a cartridge or tank. |
| **Vapor Product System, also referred to as electronic nicotine or non-nicotine delivery systems** | Refers to the specific combination consisting of the Vapor Product Refill and Vapor Product Device, which, based on the information made available to the consumer by the provider, are intended to be used together. |
| **Heated Tobacco Products (HTPs), also referred to as Heated Tobacco Product (HTP) Consumables or Heat-Not-Burn Product Consumables** | Refers to tobacco products that are intended to be consumed through heating tobacco, either electronically or through other means, sufficient to release an aerosol that can be inhaled, without combustion of the tobacco. HTP Consumables or Heat-Not-Burn Product Consumables may also include liquid solutions and gels that are part of the product and are heated to generate an aerosol. HTPs may or may not operate by means of an HTP Device. |
| **Heated Tobacco Product Device or HTP Device** | Refers to the component or combination of components of an HTP System intended to be used in combination with HTP Consumables that generate an aerosol without combustion. |
| **Heated Tobacco Product System or HTP System** | Refers to an HTP Consumable and HTP Device that are intended to be used together as a system. |
| **Refill** | Refers to a container for holding electronic liquid or Nicotine Mixture. |
| **Novel Tobacco Products** | Refer to all non-combusted substances in solid or liquid form, and innovations, either made partly of tobacco leaf as raw material or containing nicotine from tobacco, intended to be used as a substitute for cigarettes or other combusted tobacco products. |
