# Supplementary figures and images for: Examining the flavor descriptors of e-cigarettes, heated tobacco products, and nicotine pouches in the Philippines: Regulatory challenges and opportunities
Source: PLOS Glob Public Health. 2025 Feb 13;5(2):e0004248. doi: 10.1371/journal.pgph.0004248 (PMC11824988; doi:10.1371/journal.pgph.0004248)

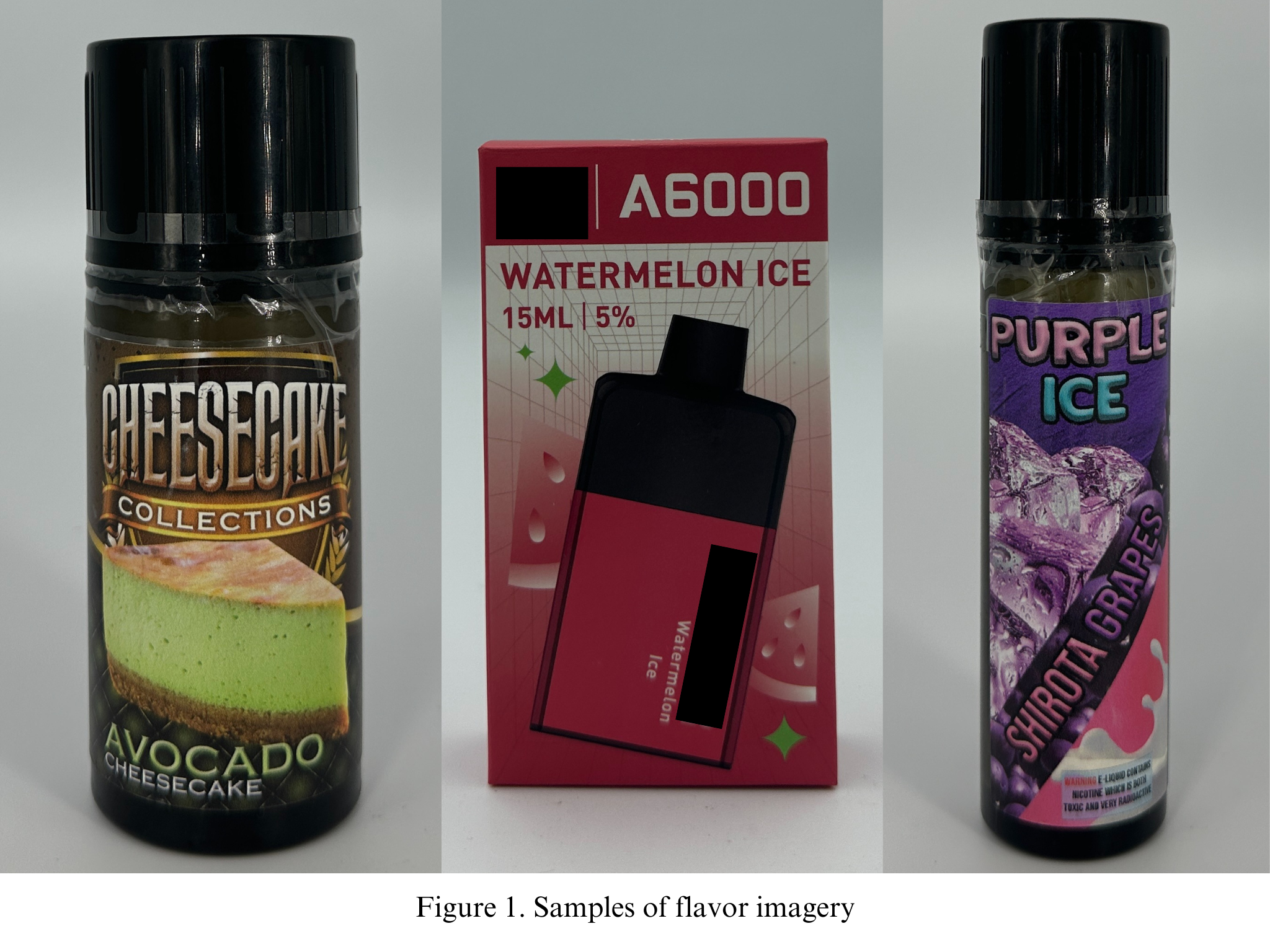

Supplement: S1 Fig — (TIFF) [file pgph.0004248.s006.tiff]

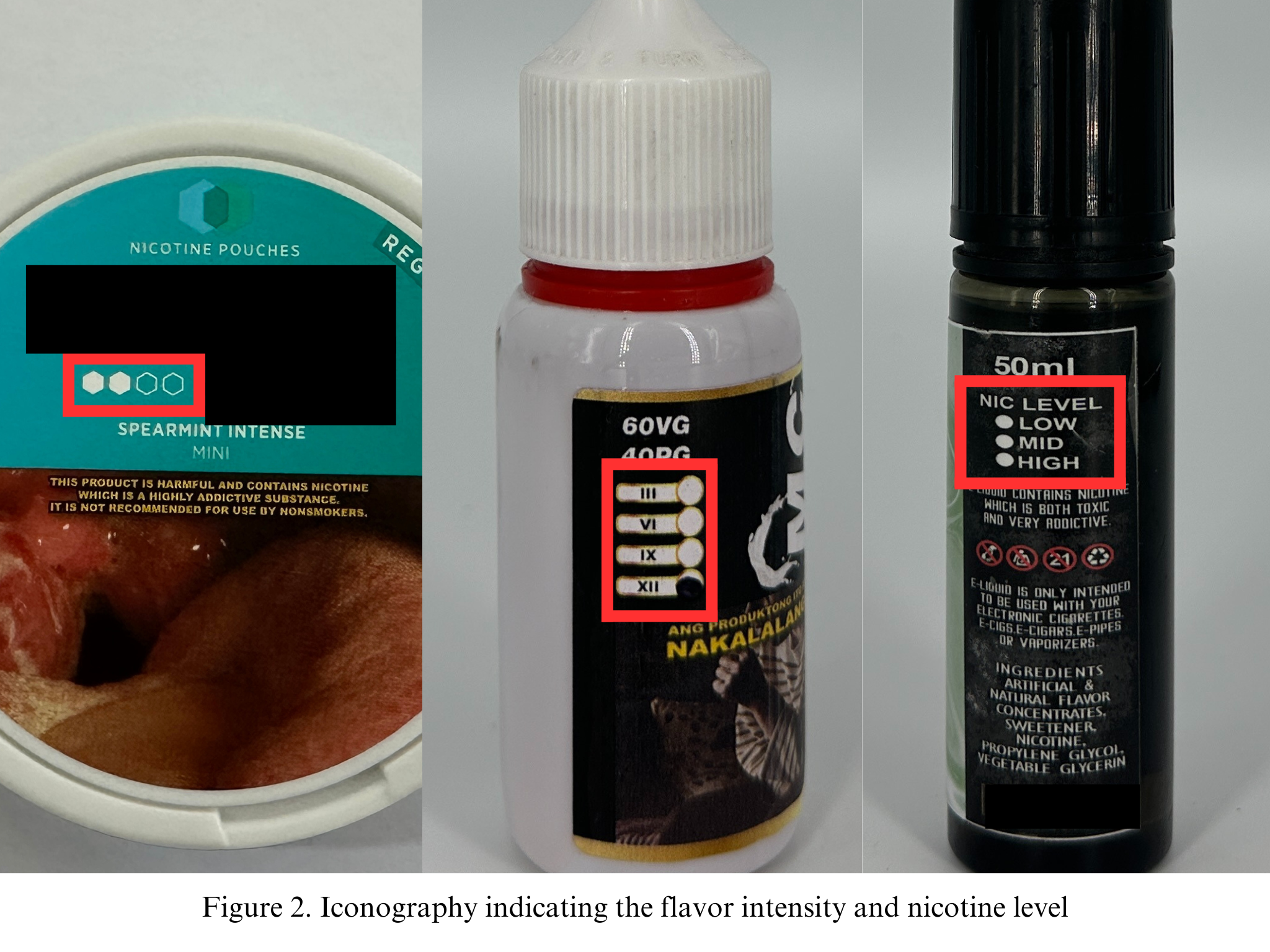

Supplement: S2 Fig — (TIFF) [file pgph.0004248.s007.tiff]

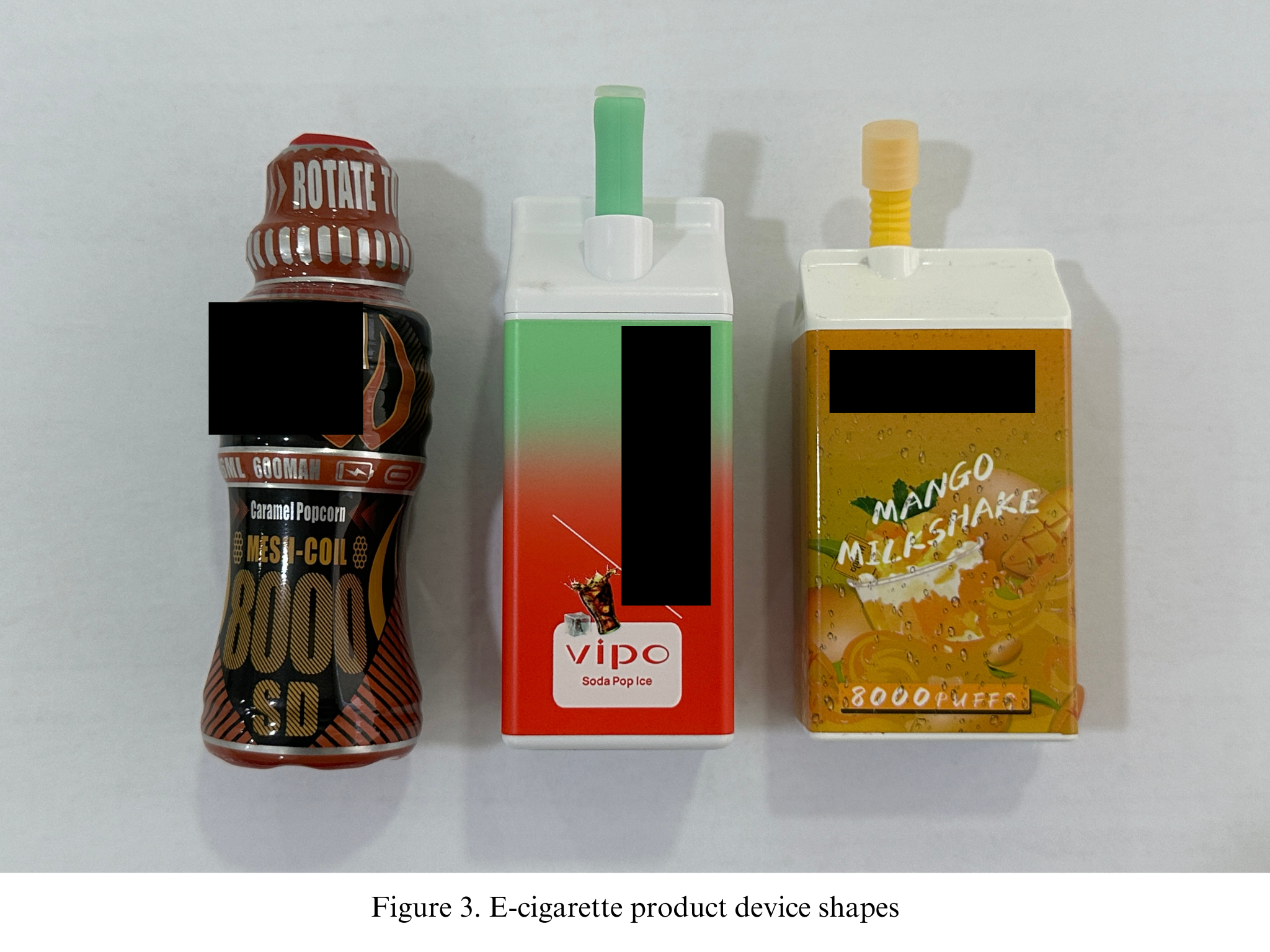

Supplement: S3 Fig — (TIFF) [file pgph.0004248.s008.tiff]

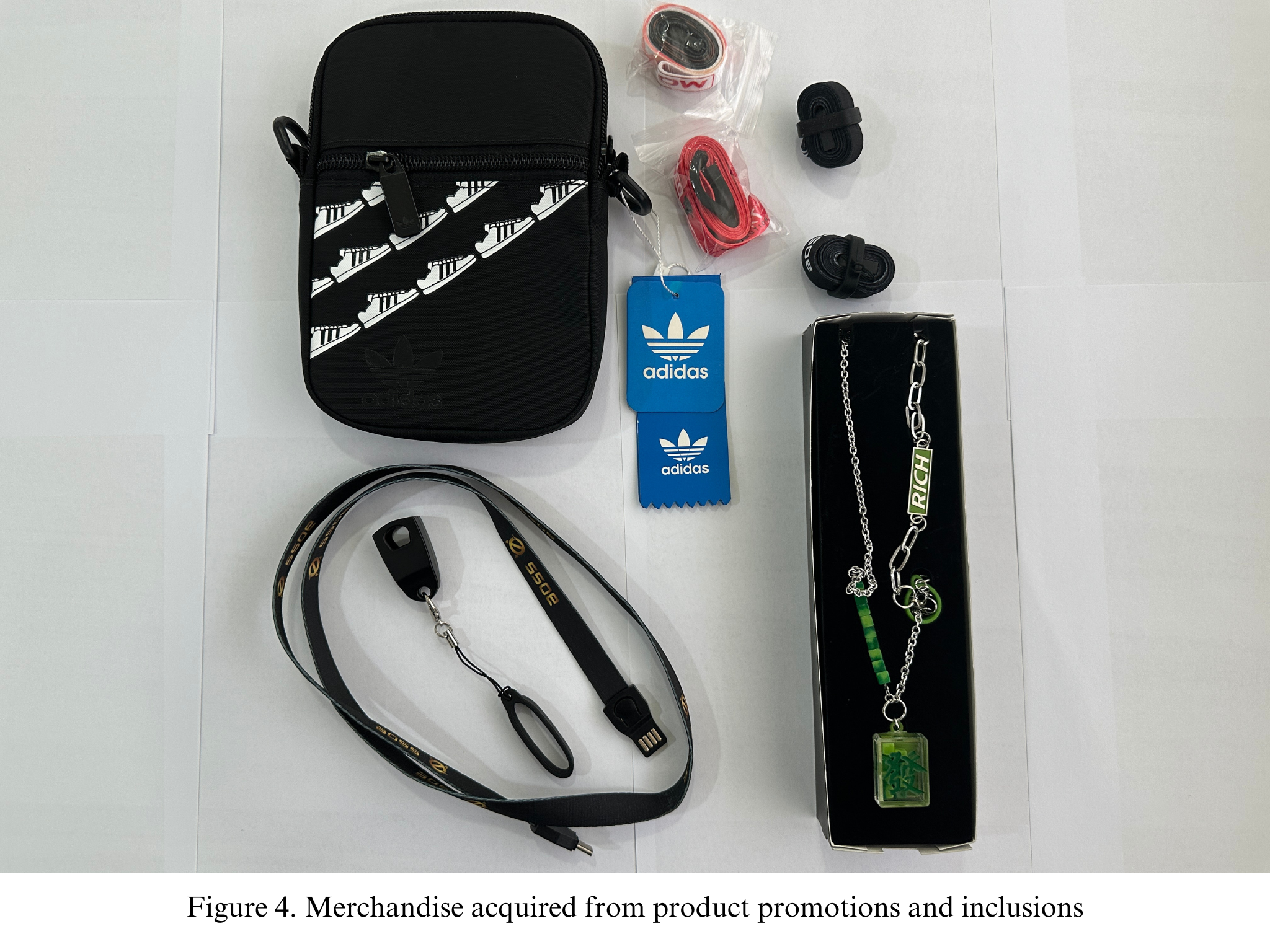

Supplement: S4 Fig — (PNG) [file pgph.0004248.s009.png]

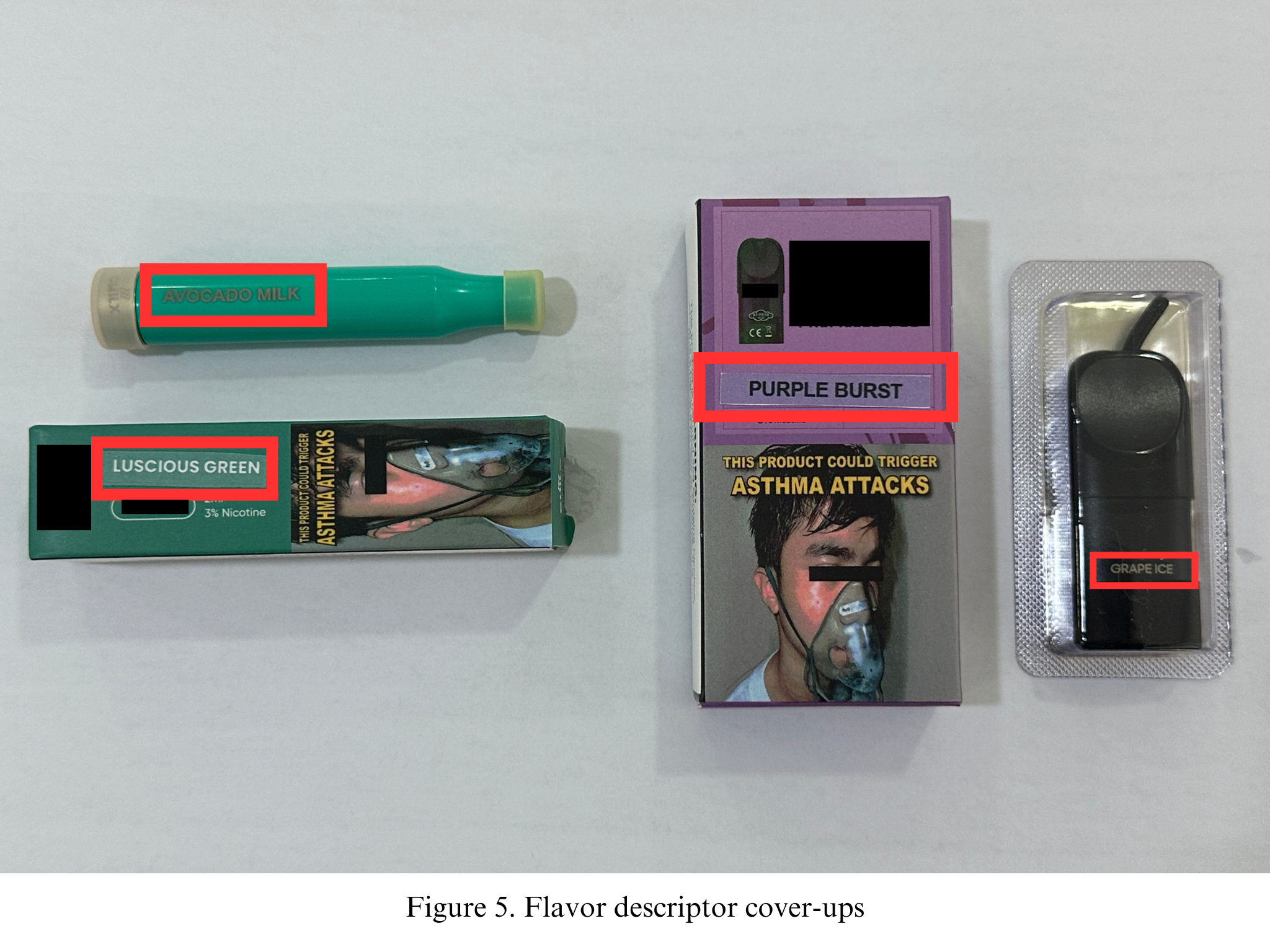

Supplement: S5 Fig — (PNG) [file pgph.0004248.s010.png]
