# Supplementary material for: Examining the flavor descriptors of e-cigarettes, heated tobacco products, and nicotine pouches in the Philippines: Regulatory challenges and opportunities
Source: PLOS Glob Public Health. 2025 Feb 13;5(2):e0004248. doi: 10.1371/journal.pgph.0004248 (PMC11824988; doi:10.1371/journal.pgph.0004248)
Supplement: Attachment — Submitted filename: Response to Reviewers.docx [file pgph.0004248.s012.docx]

**Response to Reviewers**

# Reviewer 1

Reviewer #1: While the data analysis is mainly descriptive, the study collects relevant primary data on an important public health topic, which is appropriately analyzed considering local regulations and best practices in the world. Below I provide some minor suggestions that I hope will be useful.

- Title
  - Line 3. Please consider specifying flavor descriptors/representation instead of just flavor in the title; may be more precise.
    - This is duly noted. We will revise the title to indicate “flavor descriptors” instead of just “flavors”. We will also be replacing “novel tobacco products” with “nicotine pouches” instead: “Examining the Flavor Descriptors of E-Cigarettes, Heated Tobacco Products, and Novel Tobacco Products in the Philippines: Regulatory Challenges and Opportunities” to be more precise.
- Introduction
  - Lines 37-38. Please indicate whether this increase is statistically significant (confidence intervals or a p-value would be informative).
    - Unfortunately, this data is not available.
- Methods
  - Lines 91-92 only mention cigarette and cigar packs but not ENDS, which are the focus of the analysis. Therefore, it is unclear whether only ENDS or both cigarettes and ENDS were collected. The information provided in these lines should be consistent with that in the sample section.
    - Thank you for pointing out this error. We have edited the line to the following:

Lines 249-250: “Following the TPackSS protocol, our team gathered unique e-cigarette, HTP, and nicotine pouch products from the largest city in each of the three island groups…”

- Lines 94-98. Please clarify what data were unavailable, just socioeconomic status? On lines 95-96 please be specific about the data used and the sources.
  - Thank you for highlighting the need for better clarity. We have edited the line to reflect the sources of our data and which data were unavailable:

Lines 268-280: “We chose 12 barangays or neighborhoods from each city based on specific characteristics including population density, geographical location, and economic importance (S1 Table), where we determined economic importance by identifying key economic or commercial hubs in the neighborhood, given the absence of income class data at the neighborhood level. These three characteristics were chosen to ensure logistical convenience, representativeness of the sample, and a diversity of products to access. Given that data on the socioeconomic classification was unavailable from online sources and local government authorities, we used these characteristics as proxies for socioeconomic classification. The data regarding population density, economic importance, and geographical location were based on both the Philippine Statistics Authority census results and the national tax allotment data by the Department of Budget and Management.”

- Results
  - Lines 140, 149. Information about the sample size is inconsistent: line 122 indicates 313, line 140 indicates 280 and line 149 indicates 315. On line 140 it should be specified that 280 corresponds to products with flavor descriptors.
    - Your comment is duly noted. To clarify, our team collected a total of 313 vapor products, vapor product devices, vapor product systems, heated tobacco products, heated tobacco product devices, heated tobacco product systems, refills, and novel tobacco products (line 122). However, after screening, 280 products fit into the inclusion criteria. We understand the confusion and have edited the following lines to reflect the following changes:

Line 354: “Our team collected a total of 313 vapor products…”

Lines 281-285: “The inclusion criteria of this study encompasses all products regulated by the Vape Law, which includes vapor products, vapor product systems, refills, heated tobacco products, and novel tobacco products (specifically nicotine pouches) with flavor descriptors. All the terms used for the product types in this manuscript reflect the terminology stipulated under the Vape Law (S2 Table).”

Lines 389-391: “Our team collected 313 products from 36 stores across a total of 49 barangays or neighborhoods in Quezon City, Antipolo City, Cebu City, and Davao City (S1 Table).”

- Line 142: although this is explained later in the draft, the category “codes” should include an example in parenthesis such as (e.g. acronyms).
  - Thank you for pointing this out. We understand that this will help provide more clarity on this flavor descriptor category. We have edited lines 141-142 to the following:

Lines 717-718: “In order of frequency, these 11 categories are colors, fruits, codes or acronym-like descriptors (e.g. MCM for mocha cookie milkshake), concept descriptors…”

The above passage has also been moved from the beginning of the Results section to the beginning of the Discussion section, as per the recommendation of Reviewer 3.

- Line 151. Table 1 seems unnecessary as all the information it provides is summarized on line 149 (note that the % differs, it should be the same). I suggest merging tables 1 and 2.
  - Thank you for the suggestion. We have merged Tables 1 and 2 as follows:

Table 1. Product type distribution for products with and without flavor descriptors

| **Product Type** | **Count with Flavor Descriptors** | **Count without flavor descriptors** |
| --- | --- | --- |
| Vapor Product | 70 (25.18%) | 0 (0.00%) |
| Vapor Product Device | 1 (0.36%) | 26 (74.29%) |
| Vapor Product System | 105 (37.77%) | 0 (0.00%) |
| Heated Tobacco Product | 10 (3.60%) | 6 (17.14%) |
| Heated Tobacco Product Device | 0 (0.00%) | 3 (8.57%) |
| Refill | 90 (32.37%) | 0 (0.00%) |
| Novel Tobacco Products (Nicotine pouches) | 2 (0.72%) | 0 (0.00%) |
| **Total** | **278** | **35** |

- Line 156. Why are mall kiosks the main place of purchase? Is it because this is the most common point of sale in the country? Is this result (a dominant point of sale) common when using the TPackSS walking protocol?
  - Thank you for this question. Our team found that mall kiosks often boasted a larger and more diverse range of products compared to other types of stores. This may be why our team was able to collect an extensive portion of our products from mall kiosks. In the Philippines, malling is a popular pastime amongst Filipinos, evidenced by the ubiquity of malls around the country. Generally, malls are regarded as the main place of purchase for any product; hence why we were able to acquire a large volume of our products from mall kiosks.

Moreover, we visited highly urbanized cities where numerous malls are commonly located. The TPackSS walking protocol may have positioned our team to easily come across these malls. The TPackSS protocol indicated the starting point to be a commercial hub. As such, it was a given for our team to start at malls in some, if not most, of our chosen *barangay* or neighborhood sites.

We have reflected this from the following lines in the Methodology as shown below:

Lines 441-445: “Given that malls are ubiquitous in highly urbanized cities in the Philippines, malls are regarded as key commercial hubs and the key place of purchase for any consumer product. As such, malls became a common data collection starting point for our team. We found that mall kiosks offered a large and diverse range of products compared to other types of stores. ”

- Line 162. Table 4 is very extensive and although it presents relevant information, it could be summarized by grouping by country of origin. It would also be interesting to understand how this information relates to the central point, flavor descriptors, specifically, whether there are differences (or not) in the use of flavor descriptors between domestic and foreign producers.
  - Thank you for your suggestion. We moved the original Table 4 into the Supplementary Material (now S4 Table) and replaced it with a table describing the manufacturers aggregated per country. The new table is reflected as follows:

Table 2. Manufacturer distribution by country

| **Manufacturer Country of Origin** | **Count** | **Percentage** |
| --- | --- | --- |
| **China** | 26 | 56.52% |
| **Philippines** | 12 | 26.09% |
| **Unknown** | 2 | 4.35% |
| **Japan** | 1 | 2.17% |
| **Italy** | 1 | 2.17% |
| **Hong Kong** | 1 | 2.17% |
| **USA** | 1 | 2.17% |
| **Poland** | 1 | 2.17% |
| **Indonesia** | 1 | 2.17% |
| **Total manufacturers** | 46 | 100.00% |

Additionally, we have added the following lines to relate the manufacturer information to flavor descriptors:

Lines 449-456: “Our analysis of the 280 e-cigarette products revealed that a significant majority (56.52%) originated from China, indicating the country's dominance in the e-cigarette market (Table 2). The diverse range of flavor descriptors as reflected in Table 3 suggests a strong emphasis on innovation and consumer preference, with products from the Philippines (26.09%) potentially reflecting local taste influences. Additionally, the limited representation of producers from countries like Italy and Poland points to emerging e-cigarette markets that may introduce unique flavor profiles in the Philippines.”

- Lines 219-220. This part about marketing tactics is interesting (specially the discussion in lines 301-306) but the methods do not explain that this type of information was also collected; should be specified.
  - This is noted, thank you for pointing this out. We improved the line to include this and included other information our team collected:

Lines 327-331: “Given that the initial TPackSS codebook was created for tobacco products, our team added additional elements to code for e-cigarettes, including product type based on definitions stipulated in the Vape Law (e.g. e-liquid refill, a device, etc.), the presence of nicotine, nicotine level, e-liquid capacity, and puffs. We also noted down marketing tactics such as the presence of merchandise and promotional strategies. ”

- Fig 1 to 11, if of good quality, are an interesting addition to help understand the variety of flavor descriptors. But 4 photos from each of the 11 categories may be too much. Maybe it is an editorial decision.
  - Thank you for your suggestion. Our team will keep our current format of four photos from each of the 11 categories to emphasize the richness of the data we collected.

# Reviewer 2

- Lines 40-41, “About 25% of Filipino students aged 13–15 have tried e-cigarettes while 14% currently use them” At what age can you use e-cigarettes in the Philippines? Also, is there any age confirmation at the retailers?
  - The legal age of e-cigarette use in the Philippines is 18 years old. The Vape Law lowered the age of use from 21 years old to 18 years old. The age confirmation mechanism expressed in the Vape Law is “the presentation of any valid government-issued identification card exhibiting the buyer's photograph and age or date of birth.” The presentation of this identification must be prompted by the retailer; however, from our data collection experience, there was no age verification at retailers.

We have added the following lines to emphasize the legal age of e-cigarette use.

Lines 74-79: “The prevalence of e-cigarettes use amongst adults rose from 2.8% in 2015 to 5.7% in 2021, with current users growing from 0.8% to 2.1% [3,4]. E-cigarette use is significantly higher among the youth where 25% of Filipino students aged 13–15 have tried e-cigarettes and 14% or one in seven currently use them [5]. This is alarming, considering that the current legal age for e-cigarette use in the Philippines is set at 18 years old [6].”

We have also added the following lines to describe the age confirmation required at retailers and our team’s experience in relation to it:

Lines 686-687: “The Vape Law mandates all retailers to verify the age of buyers by requiring the buyer to present any valid government-issued identification card. However, our team did not encounter any instance in which a retailer required us to present an identification card to verify our age.”

- Lines 57-60, It mentioned the Vape law, but I could not understand why the Vape law was to be enforced. If you know any reason, it would be easier to understand the story if you had. For example, political or economic reasons.
  - While purported to protect smokers, safeguard minors, generate revenue, and prevent illicit trade of the products, the Vape Law was opposed by the Philippines' Department of Health, Department of Education, various medical associations and civil society groups. This is because it has overturned several existing e-cigarette regulations and was seen to have reversed public health wins in e-cigarette control. For example, the Vape Law lowered the legal age to access these products from 21 to 18, and essentially legitimized the use of vaping products to a younger cohort.

We have included the following lines to emphasize the objectives of the Vape Law:

Lines 123-126: “In the Philippines, these products, both with and without nicotine, are currently regulated by the 2022 Republic Act 11900 or the Vape Law, which purportedly seeks to protect smokers, safeguard minors, generate revenue, and prevent illicit trade of these products [6].”

We also included the lines below to emphasize the context surrounding the implementation and enforcement of the Vapw Law:

Lines 193-195: “As a result, the Vape Law faced opposition by the Philippines’ DOH, Department of Education, and various medical associations and civil society groups for reversing wins in local e-cigarette control [24].”

- Do all e-cigarettes sold in the Philippines contain nicotine? I know there are products that do not contain nicotine, but is the legal requirement different depending on whether they contain nicotine or not? For example, age restrictions, sales locations, etc.
  - Not all e-cigarettes sold in the Philippines contain nicotine. The legal requirements do not differ for products that contain nicotine or not. We have inserted the following lines to reflect the scope of products that the Vape Law regulates:

Lines 121-126: “Hence, these findings underscore the need for effective regulations on flavored e-cigarettes, HTPs, and nicotine pouches [15,16]. In the Philippines, these products, both with and without nicotine, are currently regulated by the 2022 Republic Act 11900 or the Vape Law, which purportedly seeks to protect smokers, safeguard minors, generate revenue, and prevent illicit trade of these products [6].”

- Following up on the above question, I thought it would be informative if there were products with and without nicotine in e-cigarettes, what the proportions are with and without nicotine, and whether there is a difference in flavor, etc.
  - Thank you for the suggestion. Unfortunately, we do not have a full picture of the nicotine profiles of our products. While some products have clear indications of the nicotine level they contain, others do not. For example, some products have checkboxes for increasing dosages of nicotine, but are left unchecked, leaving us no way to confirm the true level of nicotine in the product.

We have included the passage below to share more about this:

Lines 629-637: “Iconography indicated coolness, sweetness, or nicotine intensity levels of the product, usually represented as a scale of intensity (S2 Fig). In most cases, these intensity levels are clearly and pre-indicated on the packaging. In other cases, the scale is illustrated as checkboxes that are meant to be manually checked off, in which we found some checkboxes left blank. For example, some e-liquid refills with checkboxes for nicotine levels of the product were left blank, leaving us unable to identify the nicotine dosage of the product. We also found other cases where the nicotine level is checked as either “low,” “mid,” or “high,” with no indication of the actual dosage amount for each level.

- Lines 347-349, You mentioned age-verification, but what exactly do you think you need to do to get retailers to follow your method?
  - Age-verification may be challenging to enforce. Behavioral change amongst retailers may be necessary to address. For example, offering training sessions for retailers about the legal consequences of selling to minors and the importance of compliance could help shift the mindset from seeing this as a rule to comply with. However, this may be difficult considering that a lot of retailers our team came across are e-cigarette users themselves. These training sessions could include content on the harms and risks of e-cigarette use, especially given the lack of education on such matters. This could potentially compel retailers to seriously consider strict implementation of age-verification rules.

This is reflected in the following lines as shown below:

Lines 940-963: “Enforcing age verification mechanisms necessitates a shift in retailer behavior. Targeted training sessions on the legal repercussions of selling to minors and the health risks of e-cigarette use could help achieve this. Since many retailers are users themselves, these initiatives may foster a deeper commitment to compliance and the effective implementation of age-verification practices.”

- Even though there are loopholes in the Vape law, there is still some degree of regulation, and your investigation showed that this is still not being followed. What do you think is the first step to make sure that the standards of the Vape law are followed?
  - Thank you for pointing this out. We articulated our response below:

Lines 869-880: “While the Vape Law sets regulations for vaporized nicotine and non-nicotine products, its challenges lie in its implementation. There is a need for comprehensive education amongst retailers on the stipulations of the Vape Law, along with its corresponding consequences for non-compliance. However, this cannot be achieved without enforcement bodies. One way to consider boosting enforcement is by engaging local government units and health agencies in monitoring Vape Law implementation and compliance. These bodies must conduct regular inspections and penalize violators accordingly. Efforts to achieve this are already being carried out by regional bodies such as the Metropolitan Manila Development Authority, but gaps remain in workforce capacity. Local governments should also consider introducing local task forces and volunteer brigades within communities and around schools to ensure compliance at the *barangay* level (the most basic governance level in the Philippines).”

These recommendations are reflected from lines below:

Lines 1008-1010: “Strengthening enforcement through collaboration with local government units and health agencies is crucial, which should involve regular inspections and penalties for violations.”

# Reviewer 3

Reviewer #3: Thank you for the opportunity to review your manuscript. This study provides valuable insights by adapting the TPackSS protocol to characterize flavor descriptors on e-cigarette packaging in the Philippines—a critical topic given the recent policy changes surrounding e-cigarette flavors in the country. However, the manuscript would benefit from improved structure and enhanced clarity in its methodology, as well as greater precision and consistency in the use of terminology. In particular, clearer definitions for product types and flavor descriptor categories, as well as more detailed descriptions of the methods used to examine flavor imagery, marketing strategies, and implementation observations, are needed. Below, I’ve provided specific suggestions to help improve the manuscript for your consideration.

- Abstract
  - Line 20-21: Instead of the positive adjectives, consider more precise descriptions and focus on how these products are perceived. For example, “perceived novelty” instead of “esteemed novelty”, “ever-changing flavors” instead of “innovative flavors”, “sensations that are attractive to consumers” instead of “pleasing sensations.” The Institute for Global Tobacco Control’s video on language considerations might help guide word choice: <https://youtu.be/afd-ciayb5U>
    - Thank you for sharing this resource and for your suggestions. We have edited the lines to the following, with some slight modifications due to earlier additions to the abstract:

Lines 23-27: “In the Philippines, the regulation of e-cigarettes, heated tobacco products (HTPs), and nicotine pouches under Republic Act 11900 or the Vape Law is crucial as their use continues to rise. While e-cigarettes are rapidly gaining popularity due to their social appeal, perceived novelty, ever-changing flavors, and sensations that are attractive to consumers, HTPs and nicotine pouches are continuously developing as well.”

- Line 27: It’s unclear what “codes” mean here. Some explanation would be helpful.
  - Thank you for pointing this out. To clarify this, we edited line 34 to: “...fruits (15.07%), codes or acronym-like descriptors (13.70%)...”
- There seems to be a disconnect between the results (lines 26-29) and conclusions (lines 30-34). While the study aims, methods, and results presented are around descriptors, the authors also make recommendations for imagery regulation and taxation policy. It might be helpful to add a connecting sentence between the results and conclusions for a smoother transition, and ensure all recommendations are linked to the study results presented.
  - Thank you for sharing this observation. We have included a line after line 29 as follows:

Lines 36-38: “We also examined the flavor imagery, marketing tactics, and promotional strategies used by these products. “

We also made clarifications around the taxation policy:

Lines 40-42: “Additionally, increasing taxation and implementing plain packaging can reduce the marketing appeal of these products, thereby enhancing prevention and control efforts.”

- Introduction
  - Line 36-44: Focus on e-cigarettes instead of other tobacco products. Consider removing the sentence on tobacco-related deaths (line 36) and add more on harms associated with e-cigarettes.
    - Thank you for the suggestion. We will keep the line on tobacco products, considering that this study also covers tobacco products, specifically HTPs and nicotine pouches. However, we have included additional information on HTPs and nicotine pouches, to help ground the data on tobacco-related deaths:

Lines 116-126: “Emerging risks from tobacco such as heated tobacco products (HTPs) and novel tobacco products like nicotine pouches pose a similar threat to the youth and adolescents. HTPs can attract adolescents by offering attractive flavors and sleek designs [15]. Nicotine pouch flavors are continuously developing and could contribute to the initiation and sustained usage of nicotine pouches for both youth and adults [16]. Hence, these findings underscore the need for effective regulations on flavored e-cigarettes, HTPs, and nicotine pouches [15,16]. “

We removed also added more on the harms associated with e-cigarettes:

Lines 80-88: “As e-cigarette use continues to climb, the health implications, particularly the risk of e‐cigarette or vaping‐use associated lung injury (EVALI), pose an emerging public health danger. As of 2020, 2,807 hospitalized EVALI cases and deaths have been reported to the U.S. Centers for Disease Control [7]. Likewise, Southeast Asian countries including Thailand and Malaysia have reported increasing cases of EVALI [8]. Since May 2024, the Philippines has recorded six cases of EVALI, with one of the cases being the death of a 22-year old male [8,9]. The rising prevalence of e-cigarette use, together with emerging health risks such as EVALI, emphasizes the urgent need for effective policy measures to mitigate this growing public health threat.”

- Line 45-50: This paragraph should be expanded to include a more comprehensive discussion of the literature on e-cigarette flavor appeal, particularly among youth. It would also be beneficial to reference relevant studies conducted in the region.
  - Thank you for your suggestion. We have added additional information on e-cigarette flavor appeal, and have included similar information on HTPs and nicotine pouches due to their inclusion in our study:

Lines 89-121: “The youth in Southeast Asia are vulnerable targets of e-cigarette use, especially as the e-cigarette market in the region is expected to grow by 29% by 2023, largely in Indonesia and the Philippines [10]. A wide diversity of flavors, along with trendy designs and point-of-sale promotions, are key tactics in targeting Southeast Asian youth for e-cigarette use [11]. Flavors are the most common motivation for using e-cigarettes [12]. Fruit and candy flavors are preferred and often lead to higher satisfaction and perceived addiction [13]. Additionally, flavored e-cigarettes are popular among young people because the flavors add appeal and mask the taste of nicotine [13,14]. Emerging risks from tobacco such as heated tobacco products (HTPs) and novel tobacco products like nicotine pouches pose a similar threat to the youth and adolescents. HTPs can attract adolescents by offering attractive flavors and sleek designs [15]. Nicotine pouch flavors are continuously developing and could contribute to the initiation and sustained usage of nicotine pouches for both youth and adults [16].”

- Line 46: The abbreviation “ENDS” only appears once in the whole article. Might not be needed.
  - This is noted. We have removed the abbreviation “ENDS.”.
- Lines 51-56: Rather than discussing general e-cigarette regulations, this paragraph could focus on an overview of e-cigarette flavor bans across countries, and elaborate on the associated challenges and considerations by providing more details.
  - Thank you for your suggestion. At present, only a handful of countries around the world have banned flavors in e-cigarettes, which means there is not a lot of data on the challenges of these regulations. However, we included some challenges that Findland encountered in implementing their flavor ban:

Linked 127-129: “As of 2021, 37 countries have banned e-cigarettes [17], with several countries already banning flavors. Out of 88 countries that allow the sale and regulation of e-cigarettes, at least 13 have restricted flavors other than tobacco in e-cigarettes.”

Lines 132-137: “Finland banned characterizing flavors in 2016, but enforcement faces challenges due to the wide diversity of e-cigarette products, limited resources for advancing tobacco control, and retailer reluctance to complying with stricter flavor regulations [20]. While initiating and enforcing flavor regulations can be difficult, there is a recognized need for it, especially in Asian-Pacific countries that have no e-cigarette regulations [21].”

- Line 59: Add the year for Republic Act 11900.
  - Thank you for this suggestion. We first introduced the year for Republic Act 11900 in lines 123-124: “In the Philippines, these products, both with and without nicotine, are currently regulated by the 2022 Republic Act 11900 or the Vape Law…”
- Line 61: Flavor substances are introduced here for the first time. It would be helpful to establish this concept earlier by discussing regulations on both flavor substances and descriptors in the previous paragraph when overviewing e-cigarette flavor bans. Additionally, please clarify whether the 2021 ban regulated flavor substances.
  - Thank you for pointing this out. At present, 13 countries have restricted flavors in e-cigarettes. However, it is unclear whether these restrictions include either substances, flavors, or both. We have distinguished this below:

Lines 128-131: “Out of 88 countries that allow the sale and regulation of e-cigarettes, at least 13 have restricted flavors other than tobacco in e-cigarettes. However, it is unclear whether these restrictions cover both flavorings and descriptors [18].”

We also clarified the scope of the FDA ban on flavored e-cigarettes:

Lines 179-181: “In 2022, the Philippine FDA began regulating e-cigarette flavors through FDA Administrative Order (A.O.) 2022-1069, which prohibits vapor products with flavorings or additives other than plain tobacco or menthol [22].”

- Line 63-65: This sentence is confusing and unclear. The phrase "implementation ambiguities" is not well explained. Additionally, the connection between these ambiguities and the Vape Law's repealing clause is not clearly articulated. It would be helpful if the authors clarified what they mean by "implementation ambiguities", provided a more detailed explanation of how the repealing clause contributes to these ambiguities, and included a relevant citation to support this point.
  - Thank you for pointing out the lack of clarity. We have edited the passage accordingly to highlight the connection between the ambiguities:

Lines 179-195: “In 2022, the Philippine FDA began regulating e-cigarette flavors through FDA Administrative Order (A.O.) 2022-1069, which prohibits vapor products with flavorings or additives other than plain tobacco or menthol [22]. However, the passage of the Vape Bill in 2022, now known as the Vape Law, lifted this flavor ban through its repealing clause, which nullifies previous regulations on e-cigarettes. Instead of prohibiting certain flavorings and additives, the Vape Law only prohibits “unduly appealing” flavor descriptors, or those that “include a reference to a fruit, candy brand, dessert, or cartoon character” [6]. This offered the opportunity for e-cigarette retailers to continue the distribution and sale of flavored e-cigarettes, provided that the flavor descriptors do not appeal to minors. In addition to this, the Vape Law overturned several other e-cigarette regulations such as reducing the minimum age of access from 21 years old to 18 years old; allowing the online sale and advertising of vaporized nicotine and non-nicotine products and novel tobacco products; and transferring regulatory jurisdiction from the Department of Health’s (DOH) FDA to the Department of Trade and Industry (DTI) [23]. As a result, the Vape Law faced opposition by the Philippines’ DOH, Department of Education, and various medical associations and civil society groups for reversing wins in local e-cigarette control [24].”

- Line 77-79: It is unclear what “mixed implementation” means. In addition, some elaboration on “significant opportunity to strengthen flavored and unflavored e-cigarette regulation” would be helpful.
  - Thank you once again for pointing out the lack of clarity. We have edited the lines to highlight challenges in e-cigarette policymaking and regulations, and have cited a reference that offers a recommendation into opportunities for regulation e-cigarettes.

Lines 200-231: “The DOH of the Philippines recognizes that stronger implementation is still required to further prevent and reduce e-cigarette use [27], especially in light of tobacco industry interference in policymaking for e-cigarette control [23]. Effective implementation must include evidence-based tobacco control measures, of which flavor bans must be included to protect the youth from initiating and sustaining e-cigarette use [23].”

- Line 80-82: The study cited here does categorize e-cigarette flavors. It might be more accurate to state “flavored e-cigarettes available at points of sale.”
  - Thank you for your suggestion. We have edited the lines to reflect the following statement:

Lines 232-234: “While the online marketing of e-cigarettes and heated tobacco products has been previously examined [28], no study has yet categorized the range of flavored e-cigarettes, HTPs, and novel tobacco products at points of sale in the Philippines.”

- Lines 82-83: The stated study aim is quite vague and lacks precision. It would be helpful to provide more specific details on how e-cigarette flavors will be classified (e.g., descriptors on e-cigarette packaging at points of sale in the Philippines) and which elements of the policy landscape will be assessed.
  - Thank you for pointing out the ambiguity of the stated study aim. We revised the lines as follows:

Lines 234-240: “This study aims to describe the flavor landscape e-cigarette products, HTPs, and nicotine pouches from points of sale by examining their flavor descriptors. Moreover, this study aims to examine flavor imagery that characterize these flavor descriptors, along with other strategies such as promotional and marketing tactics featured on these products. This study also outlines key implementation gaps in the Vape Law and the implications of such gaps in regulating flavored e-cigarettes, HTPs, and nicotine pouches.”

- Methods
  - Line 86: The abstract states that the TPackSS protocol was adapted, while here it says adopted. Please clarify whether the TPackSS protocol was adapted or simply adopted. If adapted, explain the modifications made. There is no need to mention TPackSS protocol repeatedly in places where it is simply followed.
    - Thank you for pointing this out. We adapted the TPackSS protocol with various modifications that consider data availability (e.g., income level of barangays) and the local economic context (e.g., economic centers). We have inserted the following lines to clarify the modifications we made:

Lines 246-247: “We made modifications to account for data unavailability (e.g. income level of *barangays*) in selecting the sites for data collection.”

Lines 274-280: “Given that data on the socioeconomic classification was unavailable from online sources and local government authorities, we used these characteristics as proxies for socioeconomic classification. The data regarding population density, economic importance, and geographical location were based on both the Philippine Statistics Authority census results and the national tax allotment data by the Department of Budget and Management.”

We also adapted the TPackSS codebook to include additional elements for coding, which we have described below:

Lines 327-331: “Given that the initial TPackSS codebook was created for tobacco products, our team added additional elements to code for e-cigarettes, including product type based on definitions stipulated in the Vape Law (e.g. e-liquid refill, a device, etc.), the presence of nicotine, nicotine level, e-liquid capacity, and puffs. We also noted down marketing tactics such as the presence of merchandise and promotional strategies.”

- Line 92-94: Four cities in each of the four regions would be 16 cities in total. Please list all 16 cities, or correct the sentence to say “from the biggest city in each region.”
  - We apologize for the confusion. Our team visited four cities in total, three from each island group of the Philippines and one from the National Capital Region. We have articulated this below:

Lines 249-268: “Following the TPackSS protocol, our team gathered unique e-cigarette, HTP, and nicotine pouch products from the largest city in each of the three island groups of the Philippines: Antipolo City (Balanced Luzon), Cebu City (Visayas), and Davao City (Mindanao). We also visited Quezon City of the National Capital Region due to its deemed economic and geographical significance.”

- Line 94-96: “We chose 12 neighborhoods (barangays) for each city based on specific characteristics like population density, economic importance, and geographical location.” Please list all the neighborhood characteristics considered (using "including" instead of "like"), specify how these characteristics informed the sampling process (e.g., was the goal to achieve balanced distribution across each parameter?), and clarify how “economic importance” was determined. In addition, it would be helpful to include the list of neighborhoods selected in the supplemental material.
  - All of the neighborhood characteristics we considered included three: population density, economic importance, and geographical location. We added the following lines as follows:

Lines 268-274: “We chose 12 barangays or neighborhoods from each city based on specific characteristics including population density, geographical location, and economic importance (S1 Table), where we determined economic importance by identifying key economic or commercial hubs in the neighborhood, given the absence of income class data at the neighborhood level. These three characteristics were chosen to ensure logistical convenience, representativeness of the sample, and a diversity of products to access.”

We have also included the list of neighborhoods in the Supplementary Material (S1 Table) and is cited accordingly as shown above.

Table 1. Selected neighborhood sites for data collection

| **Island Group** | **City** | **Barangays/Neighborhoods** |
| --- | --- | --- |
| National Capital Region | Quezon City | Kaunlaran  Bagong Pag-Asa  Socorro  Nayong Kanluran  Commonwealth  Pasong Putik  Payatas  South Triangle  Bagumbuhay  Bagumbayan  Marilag  Greater Lagro |
| Balanced Luzon | Antipolo City | Cupang  Santa Cruz  San Roque  San Luis  Bagong Nayon  Inarawan  Dalig  Beverly Hills  Dela Paz  San Jose  Mayamot  San Juan |
| Visayas | Cebu City | Santa Cruz  Lahug  Talamban  Banilad  Suba  Ermita  Mambaling  Busay  Mabolo  Lusaran  Tabunan  Luz |
| Mindanao | Davao City | Barangay 30-C  Toril  Dumoy  Lizada  Bucana  Eden  Barangay 20-B  Catigan  Talomo  Bayabas  Barangay 4-A  Tagurano |

- Lines 102-109: “Starting from a commercial hub in each neighborhood, our team walked for five minutes until we encountered one of the six specified vendor types. We then purchased one of each unique pack available for sale. […] In subsequent neighborhoods, our team purchased one of each unique pack not already obtained from previous vendors.” Please clarify “unique pack” of what.
  - According to the codebook of IGTC, a unique pack is defined as “any pack with a different design or feature, including packs differing in stick count, size, brand name presentation, colors, cellophane, and inclusion of a promotional item.” The term “pack” was used since the original TPackSS codebook was designed for tobacco products. We have adjusted the language from “pack” to “product” for better clarity. We have revised and included this line in the manuscript:

Lines 290-311: “We then purchased one of each unique product available for sale. Unique products, according to the TPackSS protocol, are products with distinct designs or features, including variations in size, brand presentation, colors, cellophane wrapping, and inclusion of promotional items.”

- Additionally, clarify how the decision was made to proceed to the next neighborhood—at what point was data collection considered complete for one neighborhood? From the description provided, it appears that data collectors only visited one store per neighborhood, which would likely not yield a representative sample across vendor types. If this is not accurate, please correct and provide additional information.
  - Thank you for pointing this out. Data collection in a neighborhood was considered complete if we were able to collect as many unique products as we could that we had not already purchased from a previous vendor. We included this line to clarify:

Lines 311-315: “In a neighborhood, we considered data collection in a neighborhood as complete if we collected as many unique products as we could that we had not already purchased from a previous vendor. In subsequent neighborhoods, our team purchased one of each unique product not already obtained from previous vendors.”

- Lines 116-118: "The codebook covers various packaging elements, including structural features (size and shape) and graphic elements (brand or flavor descriptors)." Please explicitly state the specific packaging elements examined in this study and explain how descriptors were defined.
  - Thank you for pointing out the need for better clarity. We included more information on the packaging elements that we examined:

Lines 323-331: “The codebook covers various packaging elements, including the dimensions of the package, the shape of the package, brand descriptors, and flavor descriptors. Flavor descriptors are defined as terms or phrases that convey or suggest characteristics and/or qualities in flavors of food, beverages, and others. Given that the initial TPackSS codebook was created for tobacco products, our team added additional elements to code for e-cigarettes, including product type based on definitions stipulated in the Vape Law (e.g. e-liquid refill, a device, etc.), the presence of nicotine, nicotine level, e-liquid capacity, and puffs. We also noted down marketing tactics such as the presence of merchandise and promotional strategies.”

- Lines 118-120: "Two trained researchers conducted coding in two rounds to ensure result accuracy. A third researcher joined the second round of coding to address any discrepancies identified during the initial coding sessions." Please clarify what is meant by "two rounds" of coding. How many packages were coded during each round? What do "initial coding sessions" refer to, and what steps were taken after those sessions to complete the coding of all data?
  - Thank you. We added the following lines to explain the coding process:

Lines 346-352: “We conducted two rounds of coding. Two trained researched conducted the first round of coding. The objective of the first round of coding was to assess if the sample meets the criteria for inclusion in the study, to familiarize the researcher with the samples collected, and to compile an initial list of codes. Each researcher coded all the packages included in the study. The objective of the second round of coding was to assess and validate the initial list of codes generated in the first round, with the help of a third researcher.”

- Line 122: The term “e-cigarette” is used throughout other sections of the manuscript, but the term “vapor” is introduced here. For consistency, consider using the same terminology throughout.
  - Thank you for pointing this out. We used the term “vapor” as these terms are lifted from the definitions of product types stipulated in the Vape Law. We will keep this terminology and have specified such in the below lines:

Lines 284-285: “All the terms used for the product types in this manuscript reflect the terminology stipulated under the Vape Law.”

- Lines 122-129: Please define each product type clearly and use precise terminology. For instance, if “vapor products” only include pods and cartridges, consider using “e-cigarette pods/cartridges.” If “novel tobacco products” refer solely to nicotine pouches, use “nicotine pouches” instead. Additionally, use “heated tobacco product inserts” instead of “heated tobacco products” and “e-cigarette refills” instead of “refills” for greater precision.
  - Thank you again for pointing this out. Similar to your previous concern, this manuscript reflects the terms that are defined under the Vape Law. We have emphasized this in the methods section

Lines 284-285: “All the terms used for the product types in this manuscript reflect the terminology stipulated under the Vape Law.”

- Line 129: Nicotine pouch is mentioned here for the first time. If examining nicotine pouches is part of the study aim, please state this in the introduction section and provide the rationale, including relevant background information on nicotine pouches.
  - Thank you for noting this. We have mentioned nicotine pouches in our study aim below:

Lines 232-234: “While the online marketing of e-cigarettes and heated tobacco products has been previously examined [28], no study has yet categorized the range of flavored e-cigarettes, HTPs, and novel tobacco products at points of sale in the Philippines.”

We also included additional information on the background of nicotine pouches:

Lines 119-121: “Nicotine pouch flavors are continuously developing and could contribute to the initiation and sustained usage of nicotine pouches for both youth and adults.”

- Line 126: The abbreviation “HTP” is introduced here for the first time. Please place it in parentheses following “heated tobacco product” when it is first mentioned earlier in the article. However, since lines 126 and 127 are the only places in the article where this abbreviation is used, it may not be necessary to retain it. If the authors choose to keep it, please replace “heated tobacco product” with “HTP” throughout the rest of the article for consistency.
  - This is noted. We have retained the abbreviation “HTP” as we included additional information and mentions of HTPs throughout the manuscript.
- Line 130-132: The inclusion criteria should be introduced earlier, before getting into the detailed steps of data collection.
  - Thank you for the suggestion. We have included the inclusion criteria in the Design subsection in lines below:

Lines 281-284: “The inclusion criteria of this study encompasses all products regulated by the Vape Law, which includes vapor products, vapor product systems, refills, heated tobacco products, and novel tobacco products (specifically nicotine pouches) with flavor descriptors.”

- Line 134-137: The analysis section requires significantly more detail. Please elaborate on the processes involved in the inductive and deductive approaches. Were inductive codes identified, and if so, how? Please also clarify whether the deductive approach refers to codes from the TPackSS codebook. In addition, it would be helpful to explain how flavor descriptors were defined and provide more information on the categorization process. Do the distinct codes and sub-codes refer to the codes generated through the inductive and deductive processes?
  - Thank you for pointing out the need for more detail in the analysis section. We have reflected the following edits below:

Lines 364-386: “We conducted a thematic analysis using a combination of inductive and deductive approaches. We drew deductive codes from a 2016 study conducted by the Institute for Global Tobacco Control, which identified flavor descriptor categories such as concept descriptors, menthol, beverages, and fruit or citrus [31]. Simultaneously, we used an inductive approach to identify new flavor descriptor categories not covered in the 2016 study by IGTC. This entailed reviewing the data for recurring terms and themes, leading to new categories such as pop culture and codes or acronym-like descriptors.

We first listed all flavor descriptors individually then categorized them into distinct codes and sub-codes. For example, menthol flavors were broken down into sub-codes like mint and ice. The deductive codes provided structure, while the inductive process allowed us to capture additional nuances in flavor descriptors. Finally, we synthesized these codes into overarching themes, ensuring that both predefined and newly identified descriptors were included.”

- Results
  - Line 139-147: This paragraph should be moved to the beginning of the discussion section.
    - Thank you for your suggestion. We have moved this passage to the beginning of the discussion section, now reflected in lines 733-741.
  - Line 149: Consider revising this sentence to provide a more complete statement that includes the number of stores and the locations for data collection. For example, “Our team collected 315 products from XXX stores across a total of XX neighborhoods (barangays) in Quezon City, Antipolo City, Cebu City, and Davao City. Among these, 280 (88.89%) featured flavor descriptors.”
    - We appreciate this suggestion. We have edited the line to the following:

Lines 389-394: “Our team collected 313 products from 36 stores across a total of 49 barangays or neighborhoods in Quezon City, Antipolo City, Cebu City, and Davao City (S1 Table). Among these 313 products, 280 (88.89%) featured flavor descriptors (Table 1).”

- Tables 1 & 2: These tables could be combined by listing product types in the rows and providing breakdowns by flavor descriptors in the columns. Each cell can then include numbers for counts, followed by percentages in parentheses.
  - Thank you for your suggestion. We have merged Tables 1 & 2 as reflected below:

Table 1. Product type distribution for products with and without flavor descriptors

| **Product Type** | **Count with Flavor Descriptors** | **Count without flavor descriptors** |
| --- | --- | --- |
| Vapor Product | 70 (25.18%) | 0 (0.00%) |
| Vapor Product Device | 1 (0.36%) | 26 (74.29%) |
| Vapor Product System | 105 (37.77%) | 0 (0.00%) |
| Heated Tobacco Product | 10 (3.60%) | 6 (17.14%) |
| Heated Tobacco Product Device | 0 (0.00%) | 3 (8.57%) |
| Refill | 90 (32.37%) | 0 (0.00%) |
| Novel Tobacco Products (Nicotine pouches) | 2 (0.72%) | 0 (0.00%) |
| **Total** | **278** | **35** |

- Line 156: Consider listing top 3 store types where products where purchased in the text, and remove Table 3 (or move to the appendix).
  - Thank you for your suggestion. We have moved Table 3 to the Supplementary Material (S3 Table) and edited the line to the following:

Lines 440-441: “We purchased half of the products from mall kiosks (50.71%) followed by street vendors (38.57%) and convenience stores (8.21%) (S3 Table).”

- Table 4: Consider moving this table to the appendix.
  - Thank you for your suggestion. We moved Table 4 to the Supplementary Material (S4 Table), and instead replaced it with a table listing the manufacturers aggregated per country:

Table 2. Manufacturer distribution by country

| **Manufacturer Country of Origin** | **Count** | **Percentage** |
| --- | --- | --- |
| **China** | 26 | 56.52% |
| **Philippines** | 12 | 26.09% |
| **Unknown** | 2 | 4.35% |
| **Japan** | 1 | 2.17% |
| **Italy** | 1 | 2.17% |
| **Hong Kong** | 1 | 2.17% |
| **USA** | 1 | 2.17% |
| **Poland** | 1 | 2.17% |
| **Indonesia** | 1 | 2.17% |
| **Total manufacturers** | 46 | 100.00% |

- Tables 5 & 6: Consider combining these two tables. Additionally, please use consistent terms for flavor descriptors in the tables and in the text. For example, “Fruits” is used in Table 5 and the text, whereas “Fruity” is sued in Table 6.
  - This is noted, we have combined Tables 5 and 6 and have double-checked the terms used for consistency.

Table 3. Flavor descriptor categories breakdown

| **Flavor Descriptor** | **Count** | **Percentage** | **Subcategory** | **Examples** |
| --- | --- | --- | --- | --- |
| Colors | 66 | 18.08% |  | Tangy Purple  Tropical Gold  Amber Selection  Crimson Combo  Luscious Green |
| Fruits | 55 | 15.07% |  | Strawberry Apple Banana  Passion Fruit Sunrise  Lychee Ice  Kiwi Guava  Sour Apple |
| Codes | 50 | 13.70% |  | MCM (Mocha Cookie Milkshake)  BBall (butterball)  SIC  GinPom  CADOAVO |
| Concept Descriptors | 44 | 12.05% |  | Jungle Fusion  Mellow Melody  Dreamy Swirl  Frozen oasis, chilled dopamine, tropical mist, bubble burst  Rich Roast |
| Menthol | 40 | 10.96% | Menthol  Mint  Ice | Menthol Xtra  Menthol Plus  Cool Fresh  Spearmint Intense  Minty Spears  Ice Fresh  Ice Mango |
| Beverages | 36 | 9.86% | Alcoholic beverage  Coffee  Milk  Soda  Others | Pina Colada  Lemon Mojito  Grapefruit Lime Cocktail  Latte Coffee  Choco Macchiato  Mocha Latte  Oat Milk  Cantaloupe Milk  Banana Milk Ice  Ice Cola  Orange Soda  Ribena  Pink Lemonade  Ginger Tea  Red bull sorbet |
| Dessert/Sweet/Candy | 30 | 8.22% | Dessert  Candy  Branded sweets | Mango Cheesecake  Ube brazo de mercedes  Special Halo-halo  Watermelon Candy  Grapes bubblegum  Pastillas supreme  Melona  Twinkies RY4  Dynamite Candy |
| Miscellaneous | 20 | 5.48% |  | Bazooka+  Lucid Dream  Granny's Fave  Garden's Heart  Venus Fantasy |
| Pop Culture | 13 | 3.56% | Slang words  People | Petmalu  Fucc Boi  Pink OG  Harlequin  Black Mamba  Mia Khalifa |
| Tobacco | 9 | 2.47% |  | Virginia Tobacco  Brightleaf Tobacco  Classic Tobacco  Richman's Tobacco |
| Nuts | 2 | 0.55% |  | Almond RY4  Hazelnut Brown |
| Total | 363 | 100.00% |  |  |

- Line 172: The sentence "Color descriptors are denoted as standalone colors, adjectives, or nouns" is unclear. Please clarify or rephrase for better understanding.
  - Thank you for pointing out the lack of clarity. We have edited the line as follows:

Lines 540-542: “Color descriptors can take different forms, such as simple color names (e.g. emerald), descriptive phrases (e.g. purple snow), or more imaginative terms (e.g. tropical gold) (Fig 1).”

- Line 176-177: Was the study team always able to discern what the acronym-like mean? Some descriptions on how the determinations were made would be helpful.
  - No, the study team was not always able to discern what the acronym-like codes were. We have added the following lines to describe how the determinations were made:

Lines 546-556: “We decoded the product flavors using codebooks provided by retailers, which listed flavor descriptor codes alongside their actual flavors. We also cross-checked for inconsistencies between the product packaging and the device, as there were instances where coded flavor descriptors appeared on the packaging but not on the device itself (e.g. “STRW on the packaging but “strawberry” on the device). However, identifying the flavors was not always possible, especially in cases where codebooks were unavailable.”

We have also included the following lines in the Limitations section:

Lines 1012-1016: “One limitation of our study was our reliance on retailer-provided codebooks to identify certain product flavors. In some cases, there were inconsistencies between the packaging and the device, with flavor descriptors missing from the device. Additionally, when codebooks were unavailable, we could not always identify the flavors, potentially affecting the completeness of our results.”

- Figures 1-11: Please provide figures with better image quality.
  - This is noted. We will provide figures with better image quality.
- Line 193: Please specify whether steps were taken to confirm flavor descriptors such as RY4.
  - Thank you for your suggestion. We have added lines 573-575 to address this: “To confirm the flavor descriptor for RY4, we reviewed the official websites of e-cigarette companies and retailers, where RY4 was consistently described as a blend of tobacco, vanilla, and caramel.”
- Line 194-196: How was it determined whether a descriptor is associated with a flavor, sensation, taste, or experience? Many examples in the miscellaneous category look like they could be concept descriptors.
  - We borrowed the term “concept descriptors” from the 2016 study conducted by the Institute for Global Tobacco Control, which defines concept descriptors as “terms that imply that some type of flavor, sensation, taste, or aroma awaits the consumer.” We identified key phrases such as “blast” or “fusion” that were identified in the 2016 study, which we used as basis for categorizing as concept descriptors.
- Line 209-215: The results on flavor imagery should be presented more systematically, including image examples, rather than merely listing a few examples. Additionally, please provide details on how flavor imagery was examined earlier in the methods section.
  - Thank you for the suggestion, we have revised the section as follows:

Lines 593-637: “Our team observed that visual cues play an integral role in the packaging of flavored e-cigarettes, HTPs, and nicotine pouches. In particular, images are used to connote the flavor of the product, while iconographies are used to indicate coolness, sweetness, or nicotine levels of the product.

*Imagery*

Expressive images are often used on the packaging of products in this study. Pictures of objects directly related to the flavor or flavor descriptor of the product are common. For example, ice-flavored products may feature pictures of ice cubes splashed in water, or watermelon-flavored products may feature pictures of watermelons. Images may be displayed as realistic photos of such objects, or as cartoon-like renderings (S1 Fig). Some brands offered unique flavor series (e.g., "Yakult series," a famous fermented milk drink in Asia) with related imagery (e.g. realistic images of the Yakult drinks). Disposable e-cigarettes used vibrant colors and textures, with some brands including a picture of the e-cigarette device for visualization.

*Iconography*

Iconography indicated coolness, sweetness, or nicotine intensity levels of the product, usually represented as a scale of intensity (S2 Fig). In most cases, these intensity levels are clearly and pre-indicated on the packaging. In other cases, the scale is illustrated as checkboxes that are meant to be manually checked off, in which we found some checkboxes left blank. For example, some e-liquid refills with checkboxes for nicotine levels of the product were left blank, leaving us unable to identify the nicotine dosage of the product. We also found other cases where the nicotine level is checked as either “low,” “mid,” or “high,” with no indication of the actual dosage amount for each level.”

- Line 216-220: The results on marketing strategies should be presented more systematically, including image examples. Please specify that a wide range of flavors and device shapes resembling other objects were used, instead of referring to this as “innovative marketing.” Additionally, provide details on how marketing strategies were examined earlier in the methods section.
  - Thank you for your suggestion on systematizing the results on marketing strategies. We have improved it as follows and have added image examples where relevant:

Lines 639-666: “We examined marketing strategies by noting down factors ranging from unique device shapes and mechanisms, merchandise, and social media promotion.

*Unique Device Shapes and Mechanisms*

E-cigarette devices are commonly marketed as products that look like food or beverage items. We found devices that were shaped as milk cartons, energy drinks, and juice boxes (S3 Fig). Additionally, we collected devices that have unique mechanisms, including having the ability to shift between four flavors in one device by twisting the top of the device.

*Merchandise and Brand Stretching*

The inclusion of merchandise is common, either as joint inclusions in the product box or as a freebie. The practice of brand stretching is frequent, where e-cigarette brand names and logos are used on non-e-cigarette products such as lanyards and mobile phone chargers (S4 Fig) [33]. In one instance, we received a counterfeit Adidas pouch as a freebie.

*Social Media Promotion*

Plugging social media accounts of the e-cigarette company is another common marketing tactic which involves presenting the social media handle and its corresponding platforms on the product packaging. In some cases, the verification scratch-off sticker redirects users to a website page about the product, rather than to an actual product verification page.”

Additionally, in the methods section, we included lines 330-331 on how we examined marketing strategies: “We also noted down marketing tactics such as the presence of merchandise and promotional strategies.”

- Line 221-321: The results on observations of implementation should be presented more systematically, including image examples. Additionally, please provide details earlier in the methods section on how implementation was examined, including information on the specific policies being assessed.
  - Thank you for your suggestion. We have revised this subsection and included image examples where relevant:

Lines 668-696: “Our team noted down several observations on the implementation of the Vape Law, including the presentation of flavor descriptors, age-verification mechanisms, and product testing.

*Flavor Descriptor Presentation*

The Vape Law prohibits “unduly appealing” flavor descriptors that are proven to appeal to minors, particularly if they reference a “fruit, candy brand, dessert, or cartoon character” [6]. Our team noticed inconsistencies in the presentations of flavor descriptors, which may be a response to complying with the ban on unduly appealing flavor descriptors stipulated under the Vape Law.

Some e-cigarette packs had original flavor descriptors covered by stickers with another flavor descriptor (e.g. using a sticker indicating “luscious green” to cover the original flavor descriptor “avocado milk”) (S5 Fig). In other instances, the packaging of an e-cigarette device will feature an “unduly appealing” flavor descriptor, but upon checking the device, there is a different flavor descriptor indicated (S6 Fig). For example, external packaging for a disposable e-cigarette indicated “BBALL” as the flavor descriptor, but the flavor descriptor indicated on the device was “Butterball.” Codebooks, booklets, or laminated menus of available e-cigarette flavors provided to the buyer by the sales representative, were often available to decode the true flavors for coded e-cigarette products.

*Absence of Age-Verification*

The Vape Law mandates all retailers to verify the age of buyers by requiring the buyer to present any valid government-issued identification card. However, our team did not encounter any instance in which a retailer required us to present an identification card to verify our age.

*On-The-Spot Product Testing*

In several instances, we were required to test an e-cigarette product before purchase to ensure its functionality, even though this occurred indoors in areas where e-cigarette use is prohibited under the Vape Law.”

- DISCUSSION
  - Line 238: Consider replacing “innovating flavors” with “introducing new flavors.”
    - Thank you for your suggestion. We have reflected the following changes:

Lines 730-731: “Our findings confirm that the e-cigarette industry is continuously innovating flavors beyond traditional tobacco or menthol flavors.”

- Line 239-240: Include citations for the statement that fruit, sweet, and menthol flavors typically appeal to youth and young adults.
  - Thank you for the suggestion, we have added a citation for this statement:

Lines 731-732: “We discovered flavor categories such as fruit, sweet, and menthol that typically appeal to youth or young adults [34].”

- Line 247: Avoid starting a paragraph with "For example."
  - This is duly noted. We have adjusted the statement:

Lines 747-749: “Adolescents perceive flavors such as menthol, candy, or fruit as less harmful than tobacco flavors [36], which can lead to more frequent vaping and nicotine dependence [37].”

- Line 255: Elaborate on the “rewarding and reinforcing value” of flavors.
  - We have elaborated on the definition of rewarding and reinforcing values:

Lines 754-756: “Flavors boost the rewarding value by making e-cigarettes more enjoyable and the reinforcing value by encouraging more effort to use them, increasing their addictive potential and abuse risk [41].”

- Line 258: Elaborate on “getaway drug.”
  - This is noted. We have edited the line as follows:

Lines 756-757: “Additionally, nicotine is considered a gateway drug, often leading to the use of other substances such as illegal drugs like marijuana [42].”

- Line 259: Clarify what “threshold for addiction” means.
  - We have clarified what “threshold for addiction” means:

Lines 758-759: “Using e-cigarettes with nicotine can lower the threshold for addiction to other dangerous substances, increasing susceptibility to substance dependency [43].”

- Lines 264-268: The examples provided here indicate a failure in compliance with the law rather than actual loopholes, while some of the examples discussed later point to genuine loopholes in the Vape Law. It would be beneficial to clarify this distinction in the text. Additionally, consider discussing recommendations for addressing these compliance failures separately from the required policy changes to address the loopholes.
  - To highlight this distinction, we renamed the subsection from “Challenges with implementing the Vape Law: Policy loopholes” to “Challenges with implementing the Vape Law: Compliance Gaps and Policy Loopholes.” We have highlighted the distinction in the text by adjusting the following lines:

Line 759-761: “Our team observed several compliance gaps and policy loopholes in implementing the Vape Law. One notable compliance gap is the proliferation of Vape Law-banned flavor descriptors.”

Line 819: “On the other hand, our team observed several policy loopholes in the Vape Law.”

- Line 339-342: The findings around retailer behaviors are mentioned here for the first time. These should be first introduced in the results section.
  - Thank you for noting this. We have included a line in the subsection “Observations on Implementation” that says the following:

Lines 686-689: “The Vape Law mandates all retailers to verify the age of buyers by requiring the buyer to present any valid government-issued identification card. However, our team did not encounter any instance in which a retailer required us to present an identification card to verify our age.”

- Line 350-357: The discussion around taxation appears irrelevant to the study findings.
  - We understand that the discussion around taxation may not be directly related to the findings of our study. Despite this, the prevention and control of e-cigarettes should consist of a comprehensive policy strategy that addresses not only the regulation of flavors (product packaging and branding), but also the overall availability, accessibility and affordability (e.g., price) of the product. Taxes affect price and should be considered in the policy equation for any product that contains an addictive substance in addition to taking into account the multiple ways that such products are marketed.

We included the following lines below to emphasize this point:

Lines 961-966: “While taxation may not directly relate to the findings of our study, a comprehensive policy strategy for e-cigarette prevention and control should encompass not only the regulation of flavors and branding but also the overall availability, accessibility, and affordability of e-cigarette products. With this in mind, taxes should be integral to any policy addressing both flavored and non-flavored e-cigarette products.”

- Line 382-383: More detail is required around the budgetary limitation and how it impacted the sample included in the study, which deviates from the protocol previously presented in the article.
  - Thank you for highlighting this lack of detail. We have edited lines 382-383 as follows:

Lines 1031-1035: “Additionally, due to budgetary constraints, our team was unable to purchase all unique e-cigarette products from each retailer we visited. This constraint is also amplified by the high diversity of product variations offered by retailers. However, we ensured the representation of our sample by closely following the TPackSS protocol and prioritizing the purchase of unique products from retailer to retailer.”

- Line 385: How was the representitveness of the sample assured?
  - We assured the representativeness of the sample by closely following the fundamentals of the TPackSS protocol, which includes a comprehensive sample collection strategy for accessing a diverse array of products across the country.

OVERALL

Consider using people-first language, such as people who smoke instead of smokers. (See: Hefler M, Durkin SJ, Cohen JE, Henriksen L, O'Connor R, Barnoya J, Hill SE, Malone RE. New policy of people-first language to replace 'smoker', 'vaper' 'tobacco user' and other behaviour-based labels. Tob Control. 2023 Mar;32(2):133-134. doi: 10.1136/tc-2023-057950. PMID: 36806099; PMCID: PMC9985717.)

Thank you for this insightful suggestion. We have adopted this language where relevant.

The article would benefit from a careful proofreading to correct grammatical errors.

Thank you for the suggestion. We have edited the manuscript to correct any grammatical errors.
