# Supplementary material for: Examining the flavor descriptors of e-cigarettes, heated tobacco products, and nicotine pouches in the Philippines: Regulatory challenges and opportunities
Source: PLOS Glob Public Health. 2025 Feb 13;5(2):e0004248. doi: 10.1371/journal.pgph.0004248 (PMC11824988; doi:10.1371/journal.pgph.0004248)
Supplement: Attachment — Submitted filename: Response_to_Reviewers_auresp_2.docx [file pgph.0004248.s013.docx]

# Reviewer 1

The article addresses a relevant public health issue, considering the growing use of ENDS and ENNDS, especially among young people. However, I have three general comments that I believe could improve the current draft:

1. Terminology consistency: The alternating use of "e-cigarettes" and "vapor products" is confusing, particularly since "e-cigarettes" is sometimes used to refer to all the products studied (which include HTP and nicotine pouches); e.g., lines 232 or 373-374. I believe it is standard practice to use "e-cigarettes," though it could be explained upon first mention that they are also called "vapes." The reverse approach could also work, but a single term should be used consistently throughout the text and tables, avoiding the use of "e-cigarettes" when referring to the entire sample of products.
   a. Thank you for pointing this out. Under the Vape Law, e-cigarettes are defined as “vapor products,” which include vapor products (cartridges or pods), vapor product devices (the device without any refill or cartridge), and vapor product systems (includes both the cartridge/pod and device together). We followed this terminology when categorizing the products, but we use the term “e-cigarettes” throughout the manuscript to include all products, product devices, and product systems. We have included the following clarifications to provide a better definition of our terms:

Lines 201-204: “In this manuscript, the use of the term “e-cigarettes” will collectively refer to and include all vapor products, vapor product devices, vapor product systems, and refills, in alignment with the definition under the Vape Law.”

In addition to this, we have revised the manuscript to ensure that the use of “e-cigarettes” is not used when referring to the entire sample of products.

2. Sample size and table totals: It is important to verify that the totals in the tables align with the descriptions in the text. For example:
   a. Line 218 mentions 280 featured flavor descriptors, but the corresponding column in Table 1 totals 278.
      i. Thank you for pointing out this error. We have revised the original line to the following:

Line 227: “Among these 313 products, 278 (88.89%) featured flavor descriptors (Table 1).”

b. Line 244 refers to 365 flavor descriptors, yet Table 3 shows 363.
   i. Thank you for pointing this out. We have revised the original line:

Lines 254-255: “We classified 363 flavor descriptors into seven categories…”

c. In Table 2, it is unclear why the total is 46 when the sample should include the country of origin for all products, and no missing data is reported.
   i. We apologize for the confusion. To clarify, we identified a total of 46 manufacturers across our sample of products. We have revised the line to help clarify this:

Lines 239-240: “We have identified 46 manufacturers across all samples of products (S4 Table); however, about 32.14% of products did not specify their manufacturer.”

3. Sampling Protocol: In lines 154–165, which describe the protocol for fieldwork and product purchases, it is never mentioned that not all unique products available were purchased due to budget constraints. This should be clarified in the Methods section and further elaborated on in the Limitations section (line 559), explaining how many products might have been excluded and the potential implications of this.
   a. Thank you for pointing this out. We have revised the Methods section to clarify the limitations we encountered in our data collection process:

Lines 154-164: “Starting from a commercial hub in each neighborhood, our team walked for five minutes until we encountered one of the six specified vendor types. Next, the protocol calls for the purchase of all unique products in the vendor. Unique products, according to the TPackSS protocol, are products with distinct designs or features, including variations in size, brand presentation, colors, cellophane wrapping, and inclusion of promotional items. However, in some cases, our team was unable to purchase all unique products from each vendor we visited. This is due to a limited budget and the extensive diversity particularly of e-cigarette products. In these cases, we prioritized buying at least one sample per every available brand of e-cigarette product at the vendor to minimize biases in product selection. In each neighborhood, we exhausted all available vendors in the area.”

We have also elaborated further on this in the limitations section:

Lines 571-576: “Additionally, due to budgetary constraints, our team was unable to purchase all unique products from each retailer we visited, particularly for e-cigarettes and refills. This constraint is also amplified by the high diversity of product variations offered by retailers. However, we ensured the representation of our sample by following the TPackSS protocol as closely as possible; for instance, purchasing at least one sample per every available brand of product at the vendor.”

Minor Comments:

1. Line 65: A word seems to be missing before "perceived addiction."
   a. Thank you for pointing this out. This original line as is is correct but we added “to vaping” after “perceived addiction.” This now reads:

Lines 62-63: Fruit and candy flavors are preferred and often lead to higher satisfaction and perceived addiction to vaping (13)”

2. Line 182: Replace "researched" with "researchers"?
   a. Thank you for pointing this out. We have replaced “researched” with “researchers.”
3. Line 193: Remind the reader that the inclusion criteria refer to products "with flavor descriptors”. Also, clarify in Table 1 that the column "with flavor descriptors" corresponds to the inclusion criteria.
   a. Thank you for this suggestion. We have added an additional line in the sample section to remind the reader of our inclusion criteria:

Line 198: “Our inclusion criteria covers products with flavor descriptors.”

We have also clarified in Table 1 that the column “with flavor descriptors” corresponds to the inclusion criteria:

Lines 227-230: “Among these 313 products, 278 (88.89%) featured flavor descriptors (Table 1) and fall under the inclusion criteria of this study. The majority of products with flavor descriptors are vapor product systems (37.50%), followed by refills (32.43%), vapor products (25.18%), HTPs (3.60%%), and nicotine pouches (0.72%).”

4. The title of Table 2 could be more specific about what is included.
   a. Thank you for your suggestion. We have revised the title from “Manufacturer distribution by country” to “Manufacturer distribution by country of products with flavor descriptors.”
5. The discussion about complementary policies, such as taxation and plain packaging, is relevant. However, since this is not directly related to the study results (as acknowledged in lines 515-516), please consider removing it from the abstract’s conclusions.
   a. Thank you for this suggestion. We have removed it from the abstract’s conclusions. It now reads as:

Lines 37-38: “Restoring a complete flavor ban on appealing flavors, descriptors, and imagery is crucial to reduce the marketing appeal of these products.”

6. The background in some photographs is very large.
   a. Thank you for sharing your observation. We have revised the photographs to ensure an even proportion of backgrounds.

# Reviewer #2

I was satisfied with the response to my questions. I have no further questions.

Thank you very much for your feedback.

# Reviewer #3

Thank you for addressing my suggestions. The manuscript has significantly improved, but there are still major concerns regarding the methodology, particularly related to transparency in data collection and the assessment methods. These issues must be addressed to ensure the rigor and accuracy of the study.

1. First, the methods section presents an idealized protocol, but it’s only in the limitations section that the authors reveal this protocol was not fully followed due to budget constraints and other challenges. This approach is problematic, as it misrepresents the study’s actual procedures and compromises the transparency and accuracy of the methodology. The methods section should reflect what was actually done, not only what was initially planned. Furthermore, since the team was unable to purchase all unique products, more detail is needed on how a “comprehensive sample collection strategy” was achieved. Please clarify if any specific steps were taken to reduce biases in product selection under these constraints. Without this information, it’s unclear how representative the sample can be.
   a. Thank you for pointing this out. We revised the methods section to indicate the limitations in our data collection procedure and how we addressed them:

Lines 154-164: “Starting from a commercial hub in each neighborhood, our team walked for five minutes until we encountered one of the six specified vendor types. Next, the protocol calls for the purchase of all unique products in the vendor. Unique products, according to the TPackSS protocol, are products with distinct designs or features, including variations in size, brand presentation, colors, cellophane wrapping, and inclusion of promotional items. However, in some cases, our team was unable to purchase all unique products from each vendor we visited. This is due to a limited budget and the extensive diversity particularly of e-cigarette products. In these cases, we prioritized buying at least one sample per every available brand of e-cigarette product at the vendor to minimize biases in product selection.”

2. In addition, it remains unclear how many vendors were visited in each neighborhood, and how those numbers were determined. The manuscript state, “data collection in a neighborhood was considered complete if we collected as many unique products as we could that we had not already purchased from a previous vendor.” How did the team determine that they have collected as many unique products as they could without visiting all stores? How was the definition operationalized? This also appears to contradict the statement that “due to budgetary constraints, our team was unable to purchase all unique e-cigarette products from each retailer we visited.”
   a. Thank you for pointing out your confusion. To clarify, we exhausted all available vendors in each neighborhood to reach our point of saturation. However, in some vendors that we visited, we were not able to purchase all unique products, in which case we prioritized purchasing at least one sample per every available brand of e-cigarette product at the vendor.

We have added this clarification in lines 154-164:

“Starting from a commercial hub in each neighborhood, our team walked for five minutes until we encountered one of the six specified vendor types. Next, the protocol calls for the purchase of all unique products in the vendor. Unique products, according to the TPackSS protocol, are products with distinct designs or features, including variations in size, brand presentation, colors, cellophane wrapping, and inclusion of promotional items. However, in some cases, our team was unable to purchase all unique products from each vendor we visited. This is due to a limited budget and the extensive diversity particularly of e-cigarette products. In these cases, we prioritized buying at least one sample per every available brand of e-cigarette product at the vendor to minimize biases in product selection. In each neighborhood, we exhausted all available vendors in the area.”

3. Regarding assessment, the methods section only mentions examining flavor descriptors and presence of merchandise and promotional strategies. Were flavor imagery, device shape, and compliance with the Vape Law also systematically assessed, or were those findings based on the team's ad-hoc notes? Please specify this in the methods section.
   a. Flavor imagery and device shape were noted down as ad-hoc notes. However, compliance with the Vape Law was systematically assessed based on the TPackSS codebook and existing policies regulating e-cigarette, heated tobacco, and novel tobacco products. We have another manuscript that we are finalizing for submission to a peer-reviewed journal that covers our assessment of the Graphic Health Warning compliance of e-cigarettes, heated tobacco products, and novel tobacco products with the Vape Law. We have added a line to describe this:

Lines 185-186 “Flavor imagery and device shape were recorded as ad-hoc notes while compliance of all products with the Vape Law was systematically assessed.”

4. The authors added, “To confirm the flavor descriptor for RY4, we reviewed the official websites of e-cigarette companies and retailers, where RY4 was consistently described as a blend of tobacco, vanilla, and caramel.” Was this process of confirming descriptors on official websites and checking retailer-provided codebooks consistently followed for all flavors? Please include in the methods section to clarify when and how flavor descriptors were validated.
   a. The process of confirming descriptors was only done for RY4, given that it was attached to flavor descriptors that fell under more than one category. We have added a line to clarify this:

Lines 289-292: “RY4 is a common blend of tobacco, vanilla, and caramel [32]. Given that RY4 was a recurring attachment to flavor descriptors in more than one category, we reviewed the official websites of e-cigarette companies and retailers to confirm and validate the flavor descriptor RY4.”

5. Finally, the authors added information on how the concept descriptor was defined. This should also be included in the methods section.
   a. Thank you for your suggestion. We added the definition of a concept descriptor in the Analysis section:

Lines 209-212: “We drew deductive codes from a 2016 study conducted by the Institute for Global Tobacco Control, which identified flavor descriptor categories such as menthol, beverages, fruit or citrus, and concept descriptors, which are “terms that imply that some type of flavor, sensation, taste, or aroma awaits the consumer” [31].
